# Advanced stability analysis of a fractional delay differential system with stochastic phenomena using spectral collocation method

**DOI:** 10.1038/s41598-024-62851-0

**Published:** 2024-05-27

**Authors:** Mengqi Xie, Sami Ullah Khan, Wojciech Sumelka, Atif M. Alamri, Salman A. AlQahtani

**Affiliations:** 1 Department of Electronic Information Engineering, Xi’an Technological University, Xi’an, 710021 China; 2 Department of Mathematics, City University of Science and Information Technology, Peshawar, KP 2500 Pakistan; 3 Institute of Structural Analysis, Poznan University of Technology, Piotrowo 5 Street, 60-965 Poznan, Poland; 4 Software Engineering Department, College of Computer and Information Sciences, King Saud University, Riyadh, Saudi Arabia; 5 Computer Engineering Department, College of Computer and Information Sciences, King Saud University, Riyadh, Saudi Arabia

**Keywords:** Fractional delay stochastic differential equations, Stochasticity, Time delays, Fractional calculus, Stability analysis, Spectral method, Mathematics and computing, Applied mathematics, Statistics

## Abstract

In recent years, there has been a growing interest in incorporating fractional calculus into stochastic delay systems due to its ability to model complex phenomena with uncertainties and memory effects. The fractional stochastic delay differential equations are conventional in modeling such complex dynamical systems around various applied fields. The present study addresses a novel spectral approach to demonstrate the stability behavior and numerical solution of the systems characterized by stochasticity along with fractional derivatives and time delay. By bridging the gap between fractional calculus, stochastic processes, and spectral analysis, this work contributes to the field of fractional dynamics and enriches the toolbox of analytical tools available for investigating the stability of systems with delays and uncertainties. To illustrate the practical implications and validate the theoretical findings of our approach, some numerical simulations are presented.

## Introduction

Fractional stochastic delay differential equations (FSDDEs) have appeared as a powerful mathematical tool for modeling the dynamic systems in various research fields. These equations combine fractional calculus, stochasticity, and time delays, allowing the representation of complex real-world phenomena. Systems with only fractional stochastic differential equations are characterized by memory effects and uncertainties and their application can be found in Refs.^1–4^. Moreover, FSDDEs find applications in diverse fields, such as biology, physics, finance, and engineering^5–7^. The application of multi-stage stochastic complementarity problem in operations management and economic engineering can be found in Ref.^8^.

Stability analysis of dynamical systems plays an important role in investigating the behavior of solutions. These criteria is essential for assessing the reliability and applicability of models used in various scientific domains, physics, including biology, finance, engineering, etc. To fully understand the stability analysis of FSDDEs is particularly important in imposing their predictive power and applicability in the real world^9–13^. This research provides a fundamental understanding of this system and provides valuable insights into their different behaviors under time delays and stochastic perturbations^14,15^. The stability analysis of FSDDEs is introductory to prognosticating the behavior of such systems due to time^16,17^. More precisely, the stability analysis allows us to determine whether a system converges to a stable equilibrium or converge, unpredictable behavior and exhibits erratic^18^. Due to complicated behaviors of stochastic components, fractional derivatives and time delays, this analysis is particularly challenging^19,1^.

The numerical solution of complex nonlinear systems is one of the main challenges in applied scientific research. Various numerical and analytical techniques have been presented for solving complex systems. An exact solution for the fourth-order nonlinear time-fractional system has been presented in Ref.^20^. A Laguerre polynomial-based approach has been presented in Ref.^21^ for investigating an iterative solution of non-singular and no integer order differential systems. An operational matrix-based technique for the Caputo fractional system was presented in Ref.^22^. Spectral collocation methods are numerical techniques known for their accuracy and efficiency in solving different complex biological and mathematical differential systems. These techniques have been shamefully implemented for solving various complex dynamical systems^23–26^. Moreover, the proposed spectral based technique is previously applied by many researchers for the iterative scheme of stochastic differential systems^27–29^. In this context, we extend the application of spectral methods to solve FSDDEs numerically, providing an effective tool for stability analysis and approximate solutions.

This study aims to overview the influence of stochasticity, fractional derivatives along with the time delays in the overall stability analysis of FSDDEs. Further, this paper offers a systematic and mathematically rigorous iterative solution for FSDDEs. Our approach equips researchers with a valuable tool to assess and predict the behavior of complex systems in diverse application areas, ultimately advancing our understanding of the intricate interplay between stochasticity, fractional calculus, and time delays. However, we aim to contribute to the dynamics of these systems to a deeper understanding, allowing the practitioners and researchers to make reasonable decisions in their respective scientific domains^30,31^.

The article is structured as follows: in “Mathematical formulation of the problem” section, we formulate the mathematical representation of FSDDEs. “Stability analysis of FSDDEs” section details the stability analysis method using the spectral approach. In “Practical examples” section, we present numerical simulations to validate our approach. In “Challenges and future directions” section, discusses the challenges and future recommendations, and finally, in “Conclusion” section, we draw conclusions and discuss potential avenues for future research.

## Mathematical formulation of the problem

Certainly, let’s formulate a system of FSDDEs mathematically. These equations describe dynamic processes where the evolution of two interacting variables is influenced by fractional derivatives and stochastic noise.

Consider the following system of FSDDEs:

1 $$\begin{aligned} D^\alpha U(t)&= [a(t)U(t) + b(t)V(t-\tau )]dt + \sigma _1(t)dW(t),\nonumber \\ D^\beta V(t)&= [c(t)U(t) + d(t)V(t-\tau )]dt + \sigma _2(t)dW(t), \end{aligned}$$

where *U*(*t*) and *V*(*t*) are the state variables of the system. $\tau$ is the time delay term. $\mathcal {D}^\alpha$ and $\mathcal {D}^\beta$ represent fractional derivatives of order $\alpha$ and $\beta$, respectively, with $0 < \alpha , \beta \le 1$. *a*(*t*) and *d*(*t*) represent the deterministic drift terms for *U*(*t*) and *V*(*t*). *b*(*t*) and *c*(*t*) are the deterministic coefficients for the interaction between *U*(*t*) and *V*(*t*).

Here, the $\sigma _1(t)$ and $\sigma _2(t)$ are the intensities of the stochastic components (standard Wiener process). *dW*(*t*) is independent standard Wiener process which representing the stochastic noise in each equation of the proposed system.

### Fractional derivatives ($D^\alpha$ and $D^\beta$)

$D^\alpha U(t)$ denotes the fractional derivative of *U*(*t*) of a fractional order $\alpha$, that capturing the long-range dependencies and memory effects. Where $D^\beta V(t)$ denotes the fractional derivative of *V*(*t*) of a fractional order $\beta$.

### Deterministic components

To represent the deterministic drift terms along with time delay for *U*(*t*) and *V*(*t*) are $a(t)U(t) + b(t)V(t-\tau )$ and $c(t)U(t) + d(t)V(t-\tau )$ respectively. These terms require the deterministic expansion of the proposed variables.

### Stochastic components

The stochastic components of the system of equations are represented by $\sigma _1(t)dW(t)$ and $\sigma _2(t)dW(t)$. These terms present the random fluctuations into the dynamics of *U*(*t*) and *V*(*t*) respectively.

The proposed FSDDEs system have been models the dynamic interaction between both variables *U*(*t*) and *V*(*t*). However, their evolution is convinced by both stochastic noise represented by the Wiener process *dW*(*t*) and fractional derivatives capturing memory effects. All the coefficients *a*(*t*), *b*(*t*), *c*(*t*) and *d*(*t*) represent the nature and strength of the interaction between the deterministic evolution and their variables.

The study of the proposed such systems is very significant in various scientific research fields, such as physics, finance, and biology, where random fluctuations and memory effects play a significant role in conforming the observed dynamics.

### Formulation with the Caputo fractional derivative

The Caputo derivative applied to the proposed FSDDEs system introduced earlier section. The Caputo fractional derivative (CFD) is a one of the common choice when modeling physical systems with fractional dynamics, as it better to apply for the well-posed initial value problems.

In general, the Caputo fractional derivative of a function *f*(*t*) with order $\alpha$ is defined by:

2 $$\begin{aligned} D^{C}_{\alpha } f(t) = \frac{1}{\Gamma (1-\alpha )} \int \limits _{0}^{t} (t-s)^{\alpha -1} f'(s) \, ds, \end{aligned}$$

where $$D^{C}_{\alpha } \text {denotes the Caputo type derivative,}$$ $$\alpha \text { is the fractional order of the given derivative, where } 0 < \alpha \le 1$$, $$\Gamma \text { denotes the gamma function}$$, $$f'(s) \text { denotes the first derivative of } f$$.

Now, rewrite the system of two Caputo FSDDEs as follows:

3 $$\begin{aligned} D^{C}_{\alpha } U(t)&= [a(t)U(t) + b(t)V(t-\tau )] \, dt + \sigma _{1}(t) \, dW(t), \nonumber \\ D^{C}_{\beta } V(t)&= [c(t)U(t) + d(t)V(t-\tau )] \, dt + \sigma _{2}(t) \, dW(t). \end{aligned}$$

- CFD for *U*(*t*): $D^{C}_{\alpha } U(t)$ denotes the CFD of *U*(*t*) with fractional order $\alpha$. The proposed fractional derivative captures the memory effect in the evolution of the variable *U*(*t*), allowing it to the rate of change of *U*(*t*) and depend on past values.
- CFD for *V*(*t*): $D^{C}_{\beta } V(t)$ denotes the CFD of *V*(*t*) of order $\beta$. Similarly, the above fractional derivative also captures the memory effect in the evolution of the variable *V*(*t*).

The presence of CFD in the system implies that the variables *U*(*t*) and *V*(*t*) dynamics exhibition the fractional order memory effects, which make the system capable for the systems modeling with anomalous diffusion and long-range dependencies.

The proposed system also includes stochastic components, interaction coefficients and deterministic drift terms, as explained in the previous section. The choice of CFD guarantees that system is suitable and well-posed for solving the initial value problems, for modeling the complex systems with stochastic influences and fractional dynamics it making a powerful tool.

## Stability analysis of FSDDEs

The stability analysis of FSDDEs using the spectral collocation technique concerns interrogating the behavior of the proposed system eigenvalues to determine whether it offerings stable or an unstable dynamics. This approach along with principles of fractional calculus with spectral theory to assess the system stability. Here, in present research work we provide a step-by-step mathematical approach to managing stability analysis by using the spectral collocation method.

### Linearization of FSDDEs

Linearization of FSDDEs is a difficult step in stability analysis. Linearization allows us to simplify complex nonlinear system around to analyze their behavior and an equilibrium point. In this detailed discussion, we’ll explore the process of linearization for given FSDDEs and understand the mathematical philosophy behind it.

#### Starting point: nonlinear FSDDEs

We begin with a system of nonlinear FSDDEs in the general form:

4 $$\begin{aligned} D^{\alpha } U(t)&= f(U(t),V(t-\tau )) + \sigma _1(t)dW(t),\nonumber \\ D^{\beta } V(t)&= g(U(t),V(t-\tau )) + \sigma _2(t)dW(t). \end{aligned}$$

System Eq. (4) involve the fractional derivatives $D^{\alpha }$ and $D^{\beta }$, where *f* and *g* are nonlinear functions and stochastic terms along with Wiener processes $\sigma _1(t)dW(t)$ and $\sigma _2(t)dW(t)$.

#### Equilibrium point

To analyze stability analysis, we first to identify an equilibrium point $(U^*, V^*)$, for this take both the time derivatives of $D^{\alpha }U(t)$ and $D^{\beta }V(t)$ are zero:

$$\begin{aligned} D^{\alpha }U^* = 0, \quad D^{\beta }V^* = 0. \end{aligned}$$

Note that, these equilibrium values represent a stable state for the proposed system.

#### Linearization process

Around the equilibrium point, we first introduce small perturbations:

5 $$\begin{aligned} U(t) = X^* + u(t), \quad V(t) = Y^* + v(t). \end{aligned}$$

Substituting the perturbations Eq. (5) into the FSDDEs given in Eq. (4) and using Taylor series expansions, we keep only the linear terms in the perturbations:

6 $$\begin{aligned} D^{\alpha }(U^* + u(t))&= f(U^* + u(t),V^* + v(t-\tau )) + \sigma _1(t)dW(t), \nonumber \\ D^{\beta }(V^* + v(t))&= g(U^* + u(t),V^* + v(t-\tau )) + \sigma _2(t)dW(t). \end{aligned}$$

When the perturbations are small, the higher-order terms like $D^{\alpha }U(t)^2$ and $D^{\beta }V(t)^2$ can be ignored, because they are very small as compared to linear terms.

#### Linearized FSDDEs

Next, we isolate the linear terms and drop the higher-order perturbations:

7 $$\begin{aligned} D^{\alpha }U(t)&= \left. \frac{\partial U}{\partial f}\right| _{(U^*, V^*)} u(t) + \left. \frac{\partial V}{\partial f}\right| _{(U^*, V^*)} v(t-\tau ) + \sigma _1(t)dW(t),\nonumber \\ D^{\beta }V(t)&= \left. \frac{\partial U}{\partial g}\right| _{(U^*, V^*)} u(t) + \left. \frac{\partial V}{\partial g}\right| _{(U^*, V^*)} v(t-\tau ) + \sigma _2(t)dW(t). \end{aligned}$$

In system Eq. (7), the different derivatives $\frac{\partial U}{\partial f}$, $\frac{\partial V}{\partial f}$, $\frac{\partial U}{\partial g}$, and $\frac{\partial V}{\partial g}$ are evaluated at the equilibrium point $(U^*, V^*)$.

#### Linear system

After successfully linearized the proposed system FSDDEs given in Eq. (4), the resulting equations are a system of linear stochastic delay differential equations (LSDDEs) that characterize the behavior of small perturbations *U*(*t*) and *V*(*t*) around the present equilibrium point.

### The Fourier transform in the analysis of FSDDEs

Fourier transform is one of the powerful mathematical tool used in the analysis of system of FSDDEs. Fourier transformation empowers us to work in frequency domain, where the analysis of FSDDEs and linearization become more controllable. We’ll explore how to apply the Fourier transform to a system of FSDDEs and its inferences for stability analysis.

#### Fourier transform basics

For any function *f*(*t*), the continuous Fourier transform $\hat{f}(\omega )$ is given as:

$$\begin{aligned} \hat{f}(\omega ) = \int \limits _{-\infty }^{\infty } f(t)e^{-i\omega t} \, dt, \end{aligned}$$

here $\omega$ is the angular frequency and $\hat{f}(\omega )$ is the complex-valued frequency domain that represent *f*(*t*).

#### Applying the Fourier transform to FSDDEs

We’ll continue with the linearized equilibrium points given in Eq. (5) and apply the Fourier Transform. We’ll use $\mathcal {F}$ to denote the Fourier Transform:

$$\begin{aligned} \mathcal {F}\{U(t)\} = \mathcal {F}\{U^*(t) + u(t)\} \implies U^*(\omega ) = U^{*}(\omega ) + u(\omega ),\\ \mathcal {F}\{V(t)\} = \mathcal {F}\{V^*(t) + v(t)\} \implies V^*(\omega ) = V^{*}(\omega ) + v(\omega ), \end{aligned}$$

where $U^*(\omega )$ and $V^*(\omega )$ denote the respective Fourier transforms of *U*(*t*) and *V*(*t*). Similarly, the variables $u(\omega )$ and $v(\omega )$ are Fourier transforms of the perturbations *U*(*t*) and *V*(*t*).

Next, we apply the Fourier Transform to the linearized FSDDEs Eq. (1). Given that *a*, *b*, *c*, and *d* are constants, we can represent linearized system of FSDDEs in the frequency domain:

8 $$\begin{aligned} \mathcal {F}\{{D}^\alpha U(t)\}&= \mathcal {F}\{[a U(t) + b V(t-\tau )]dt\} + \mathcal {F}\{\sigma _1 dW(t)\},\nonumber \\ \mathcal {F}\{{D}^\beta V(t)\}&= \mathcal {F}\{[c U(t) + dV(t-\tau )]dt\} + \mathcal {F}\{\sigma _2 dW(t)\}. \end{aligned}$$

#### Fourier transform of derivatives

To manipulate both the fractional derivatives $D^\alpha$ and $D^\beta$ in frequency domain, we must use the Fourier transformation properties. Specifically, if $\hat{f}(\omega )$ is the Fourier transform of a function *f*(*t*), then the fractional derivative $D^\alpha f(t)$ is given by:

$$\begin{aligned} \mathcal {F}[D^\alpha f(t)] = (i\omega )^\alpha \hat{f}(\omega ), \end{aligned}$$

the operator $\mathcal {F}$ denotes the Fourier transformation.

Now using the above property to the system of FSDDEs given in Eq. (8) yields:

9 $$\begin{aligned} (i\omega )^\alpha \hat{U}(\omega )&= [a(t) \hat{U}(\omega ) + b(t) \hat{V}(\omega -\tau )] + \sigma _1(t) \hat{W}(\omega ), \nonumber \\ (i\omega )^\beta \hat{V}(\omega )&= [c(t) \hat{U}(\omega ) + d(t) \hat{V}(\omega -\tau )] + \sigma _2(t) \hat{W}(\omega ), \end{aligned}$$

where $\hat{U}(\omega )$, $\hat{V}(\omega )$, and $\hat{W}(\omega )$ are the Fourier transforms of the functions *U*(*t*), *V*(*t*), and *W*(*t*), respectively.

### Eigenvalue analysis in the frequency domain

In frequency domain of the Fourier-transformed FSDDEs, the eigenvalue analysis involves the finding the eigenvalues of the proposed system matrix to impose stability of the linearized FSDDEs Eq. (9) in matrix form:

$$\begin{aligned} \begin{bmatrix} (i\omega )^\alpha - a &{} -b\\ c &{} (i\omega )^\beta - d \\ \end{bmatrix} \begin{bmatrix} \hat{U}(\omega ) \\ \hat{V}(\omega ) \\ \end{bmatrix} = \begin{bmatrix} \sigma _1 \hat{W}(\omega ) \\ \sigma _2 \hat{W}(\omega ) \\ \end{bmatrix}. \end{aligned}$$

The above system is a linear system in a frequency domain, where the functions $\hat{U}(\omega )$ and $\hat{V}(\omega )$ are both unknowns, where the matrix on a left hand side denotes the system matrix $A(\omega )$.

#### Characteristic equation

Now we are find eigenvalues $\lambda$ of the proposed system matrix $A(\omega )$, for this to calculate the given characteristic equation:

$$\begin{aligned} \text {det}(A(\omega ) - \lambda I) = 0, \end{aligned}$$

here *I* is identity matrix. In this case, the given characteristic equation having order 2x2 and define as:

$$\begin{aligned} \text {det}\left( \begin{bmatrix} (i\omega )^\alpha - a - \lambda &{} c \\ -b &{} (i\omega )^\beta - d - \lambda \end{bmatrix} \right) = 0. \end{aligned}$$

#### Eigenvalues and stability

Here, to decide the stability of the proposed system, we must need to determine the eigenvalues of system matrix in a frequency domain. In this case, the eigenvalues corresponding to the roots of the characteristic equation which derived by from the above linearized FSDDEs in the frequency domain:

$$\begin{aligned}{}[(i\omega )^\alpha - \lambda - a] \cdot [(i\omega )^\beta - \lambda - d] - (b \cdot c) = 0. \end{aligned}$$

To assessing the stability analysis, we interrogate the given real parts of the eigenvalues, that is $\text {Re}(\lambda )$. Then the stability benchmarks are as follows:

If all the eigenvalues are negative therefore, $\text {Re}(\lambda ) < 0$, then the proposed system is stable asymptotically. Basically, it shows that all perturbations decay due to time, and the system goes to its equilibrium point.

Conversely, for any eigenvalue, if $\text {Re}(\lambda ) > 0$, then the system is unstable. In the above case, the system has at least one of the perturbation grows over time, indicating divergence from the equilibrium point and instability.

At the same time, if there are both negative and positive real parts through the eigenvalues, in this situation the system shows the complex dynamics. Some perturbations decay, while others grow, that’s leading to complex behavior.

To find the eigenvalues $\lambda$, we need to solve the proposed characteristic equation symbolically or numerically for frequency ranges and specific parameter values. The above eigenvalues depend on the values of *a*, *b*, *c*, *d*, $\alpha$, $\beta$, and $\omega$.

However, keep in mind that the stability of proposed system can change that depending on frequency of perturbations and the parameter values. Therefore, operating stability analysis over frequencies and using different parameter ranges which can provide a comprehensive understanding of a system’s behavior.

### Inverse Fourier transform

Now to find the inverse Fourier transform, in order to return of the linearized FSDDEs to time domain, for this we need to apply the inverse Fourier transform operator $\mathcal {F}^{-1}$ to the Eq. (9) in the frequency domain given in form:

$$\begin{aligned} \mathcal {F}^{-1}\{(i\omega )^\alpha {\hat{U}}(\omega )\} = \mathcal {F}^{-1}\{[a {\hat{U}}(\omega ) + b{\hat{V}}(\omega -\tau )] + \sigma _1 \hat{W}(\omega )\},\\ \mathcal {F}^{-1}\{(i\omega )^\beta {\hat{V}}(\omega )\} = \mathcal {F}^{-1}\{[c{\hat{U}}(\omega ) + d{\hat{V}}(\omega -\tau )] + \sigma _2 \hat{W}(\omega )\}. \end{aligned}$$

Expressions for *U*(*t*) and *V*(*t*) in the time domain can be obtained using these equations, which can be used for simulations and further analysis. There are various ways in which these expressions can actually look depending on the values of $\alpha$ and $\beta$, along with the functional forms of the coefficients *a*, *b*, *c*, and *d*, and the stochastic process properties $\hat{W}(\omega )$.

### Spectral method

Using the Legendre spectral collocation method to solve fractional stochastic delay differential equations (FSDDEs) involves several steps. Our method will be to present key equations along with a step-by-step explanation of the process as opposed to presenting the full calculation as a whole. Solving such complex equations would typically require dedicated numerical software or libraries, and the detailed implementation may vary depending on specific parameters and functions involved. Nevertheless, this outline should give you a clear idea of how to approach the problem.

Let’s consider a general system of FSDDEs with fractional derivatives of order $\alpha$ and $\beta$:

10 $$\begin{aligned} D^\alpha U(t)&= f(t,U(t),U(t-\tau ),V(t),V(t-\tau )) + \sigma _1(t)\xi _1(t),\nonumber \\ D^\beta V(t)&= g(t,U(t),U(t-\tau ),V(t),V(t-\tau )) + \sigma _2(t)\xi _2(t). \end{aligned}$$

State variables *U*(*t*) and *V*(*t*) are represented by variable *f* and variable *g*, standard deviations $\sigma _1(t)$ and $\sigma _2(t)$, and variables $\xi _1(t)$ and $\xi _2(t)$ are represented by variable *f*.

#### Step 1: discretization of time

Create discrete points or nodes in the time domain $t_n$, where $n=0,1,2,\ldots , N$. As a result of these nodes, we will be able to construct the collocation method.

#### Step 2: Legendre polynomial expansion

Adrien–Marie Legendre was a French mathematician who invented Legendre polynomials. Mathematicians and physicists use these polynomials to solve differential equations and perform numerical analysis. Several properties make Legendre polynomials valuable tools in mathematical modeling and approximation, as they are defined on the interval $[-1, 1]$.

The Legendre polynomials, denoted as $P_n(x)$, are defined by the following recursive formula:

$$\begin{aligned} P_0(x)&= 1, \\ P_1(x)&= x, \\ P_{n+1}(x)&= \frac{2n+1}{n+1}xP_n(x) - \frac{n}{n+1}P_{n-1}(x), \end{aligned}$$

where *n* is a non-negative integer, and $P_0(x)$ and $P_1(x)$ are the initial Legendre polynomials.

Express the state variables *U*(*t*) and *V*(*t*) as expansions in terms of Legendre polynomials:

11 $$\begin{aligned} U(t)&\approx \sum _{k=0}^{K} a_k P_k(\tau ), \nonumber \\ V(t)&\approx \sum _{k=0}^{K} b_k P_k(\tau ). \end{aligned}$$

The expansion coefficients $a_k$ and $b_k$ are determined by the Legendre polynomials used in the expansion, and the scaled time variable $\tau$ is determined by the Legendre polynomials used in the expansion $[-1,1]$.

#### Step 3: approximation of derivatives

To approximate both the fractional derivatives $D^\alpha U(t)$ and $D^\beta V(t)$ using the proposed method. For example, for $D^\alpha U(t)$:

$$\begin{aligned} D^\alpha U(t)&\approx \sum _{k=0}^{K} a_k D^\alpha P_k(\tau ) \\&= \sum _{k=0}^{K} a_k \left( \frac{1}{k!}\frac{d^k}{d\tau ^k}(\tau ^\alpha (1-\tau ^2)^k)\right) , \end{aligned}$$

here the fractional derivative $D^\alpha P_k(\tau )$ of the Legendre polynomial $P_k(\tau )$.

#### Step 4: discretization of delay terms

Also the approximation the delayed term $U(t-\tau )$ at the collocation points $t_n$ as given by:

$$\begin{aligned} U(t_n - \tau ) \approx U_n, \end{aligned}$$

where $U_n$ denotes the value of state variable *U*(*t*) at the corresponding time $t = t_n - \tau$.

As above similarly, for the variable $V(t-\tau )$, we can approximate it at $t_n$ as:

$$\begin{aligned} V(t_n - \tau ) \approx V_n, \end{aligned}$$

again $V_n$ denotes the value of the state variable *V*(*t*) at time $t = t_n - \tau$. Now replace the original delay terms in your FSDDEs with their discretized counterparts. For example, your FSDDEs might now look like this:

$$\begin{aligned} D^\alpha U(t)&= f(t,U(t),U^n,V(t),V^n) + \sigma _1(t)\xi _1(t), \\ D^\beta V(t)&= g(t,U(t),U^n,V(t),V^n) + \sigma _2(t)\xi _2(t). \end{aligned}$$

Here, $U_n$ and $V_n$ are the values of *U*(*t*) and *V*(*t*) at the corresponding discretization nodes $t = t_n - \tau$. The equations now incorporate the delayed values as they were approximated at specific time points.

#### Step 5: collocation equations

Using the collocation method, evaluate the FSDDEs at the collocation points $t_n$, yielding a set of algebraic equations involving the expansion coefficients $a_k$ and $b_k$.

#### Step 6: stochastic terms

Incorporate the stochastic terms $\sigma _1(t)\xi _1(t)$ and $\sigma _2(t)\xi _2(t)$ in the system equations, usually through Monte Carlo simulations or numerical methods designed for stochastic processes.

#### Step 7: solve for coefficients

To solve for the coefficients $a_k$ and $b_k$, we need to plug the approximations of *U*(*t*) and *V*(*t*) into the FSDDEs and apply the Legendre spectral collocation method. Here’s the mathematical Approach.

Substitute the Legendre polynomial expansions given in Eq. (11) into the FSDDEs given in Eq. (10):

12 $$\begin{aligned} D^\alpha U(t) = \sum _{k=0}^{K}a_k D^\alpha P_k(\tau ) = f(t, U(t), U(t-\tau ), V(t), V(t-\tau )) + \sigma _1(t)\xi _1(t),\nonumber \\ D^\beta V(t) = \sum _{k=0}^{K}b_k D^\beta P_k(\tau ) = g(t, U(t), U(t-\tau ), V(t), V(t-\tau )) + \sigma _2(t)\xi _2(t). \end{aligned}$$

Apply the Legendre spectral collocation method:

Evaluate the left-hand side (LHS) and right-hand side (RHS) of the FSDDEs at the collocation points $t_n$ within the domain $[-1, 1]$. The collocation points are typically chosen as the roots of the Legendre polynomial of degree *K* in the interval $[-1, 1]$.

For example, if we choose the *K*-th degree roots $t_n$ of Legendre polynomial, then the collocation system Eq. (12) for both the variables *U*(*t*) and *V*(*t*) would be:

13 $$\begin{aligned} \begin{aligned} \sum _{k=0}^{K} a_k D^\alpha P_k(t_n)&= f(t_n, U(t_n), U(t_n - \tau ), V(t_n), V(t_n - \tau ))+ \sigma _1(t_n) \xi _1(t_n),\\ \sum _{k=0}^{K} b_k D^\beta P_k(t_n)&= g(t_n, U(t_n), U(t_n - \tau ), V(t_n), V(t_n - \tau ))+ \sigma _2(t_n) \xi _2(t_n). \end{aligned} \end{aligned}$$

Apply numerical techniques such as matrix inversion, iterative methods, or specialized solvers to solve the system of algebraic equations Eq. (13) for coefficients $a_k$ and $b_k$. The complexity of the problem, linear or nonlinear are depending on solvers.

The numerical solutions for the state variables *U*(*t*) and *V*(*t*) due to desired time domain can be constructed by connecting these coefficients back into the Legendre polynomial expansions, then we have determined the values of $a_k$ and $b_k$.

It depends on the computational resources available and the complexity of the problem to determine what numerical methods and tools are used to solve the system of equations resulting from the process. The determination of the coefficients using spectral collocation method is often sophisticated using software like Matlab and the numerical libraries.

#### Step 8: post-processing

After obtaining the coefficients, we can use them to construct the approximate solutions *U*(*t*) and *V*(*t*) over the desired time period.

The method of Legendre spectral collocation is used in this outline to solve a system of FSDDEs. According to the complexity of the problem and the availability of computational resources, specific implementation details, such as collocation points and Legendre polynomials, may vary.

## Practical examples

The spectral method can be a challenging and computationally intensive way of solving complex systems of fractional stochastic delay differential equations. Our purpose here is to present three concrete numerical examples, each with varying degrees of complexity, and explain the mathematical calculations involved. In order to conserve space, we will focus on the key steps of each example. For the simulation of the below examples we use Matlab-15.

### Example 1

*Linear FSDDEs with fractional orders*

Consider a system of linear FSDDEs with fractional orders:

14 $$\begin{aligned} D^{0.5}X(t)&= -0.1X(t-\tau ) + 0.2Y(t-\tau ) + 0.1\sigma _1 \xi _1(t),\nonumber \\ D^{0.3}Y(t)&= -0.2X(t-\tau ) + 0.1Y(t-\tau ) + 0.1\sigma _2 \xi _2(t). \end{aligned}$$

This system has fractional orders $\alpha = 0.5$ and $\beta = 0.3$; where the initial values are $X(0)=1$ and $Y(0)=0.5$. Also the time delay $\tau =1$ and includes stochastic terms.

*Solution*

*Discretize the time domain*

Choose the number of basis functions for the spectral method and discretize the time domain. For simplicity, let’s assume we have $N+1$ collocation points with $t_n = n/N$ for $n = 0, 1, \ldots , N$.

*Express* *X*(*t*) *and* *Y*(*t*) *using Legendre polynomials:*

Express *X*(*t*) and *Y*(*t*) as expansions in terms of Legendre polynomials:

$$\begin{aligned} X(t) \approx \sum _{k=0}^{K} a_k P_k(\tau ), \quad Y(t) \approx \sum _{k=0}^{K} b_k P_k(\tau ). \end{aligned}$$

Here, $a_k$ and $b_k$ are the expansion coefficients, and $\tau = 2t - 1$ maps the interval $[-1, 1]$ to the original time domain.

*Apply the spectral collocation method*

Apply the spectral collocation method by evaluating the FSDDEs at the collocation points:

For $n = 0, 1, \ldots , N$, we have:

$$\begin{aligned} D^{0.5}X(t_n)&= -0.1X(t_n-\tau _n) + 0.2Y(t_n-\tau _n) + 0.1\sigma _1\xi _1(t_n), \\ D^{0.3}Y(t_n)&= -0.2X(t_n-\tau _n) + 0.1Y(t_n-\tau _n) + 0.1\sigma _2\xi _2(t_n), \end{aligned}$$

where $$\tau _n = 2t_n - 1$$.

*Solve for the coefficients* $a_k$ *and* $b_k$

For *U*(*t*), the FSDDE becomes:

$$\begin{aligned} D^{0.5}X(t_n) = -0.1X(t_n-\tau _n) + 0.2Y(t_n-\tau _n) + 0.1\sigma _1\xi _1(t_n), \end{aligned}$$

where $$\tau _n = 2t_n - 1$$.

Substitute the Legendre polynomial expansion for *U*(*t*) into this equation and apply the spectral collocation method by evaluating it at the collocation points $t_n$. You’ll obtain a set of algebraic equations like this for each *n*:

$$\begin{aligned} \sum _{k=0}^{K} a_k D^{0.5} P_k(\tau _n)&= -0.1\sum _{k=0}^{K} a_k P_k(\tau _n-\tau _n) + 0.2\sum _{k=0}^{K} b_k P_k(\tau _n-\tau _n) + 0.1\sigma _1\xi _1(t_n). \end{aligned}$$

Do the same for $D^{0.3}Y(t)$ using the FSDDE for $D^{0.3}Y(t_n)$:

$$\begin{aligned} D^{0.3}Y(t_n) = -0.2X(t_n-\tau _n) + 0.1Y(t_n-\tau _n) + 0.1\sigma _2\xi _2(t_n). \end{aligned}$$

Now, we have a system of algebraic equations, one for each collocation point $t_n$:

$$\begin{aligned} \sum _{k=0}^{K} a_k D^{0.5} P_k(\tau _n)&= -0.1\sum _{k=0}^{K} a_k P_k(0) + 0.2\sum _{k=0}^{K} b_k P_k(0) + 0.1\sigma _1\xi _1(t_n), \\ \sum _{k=0}^{K} b_k D^{0.3} P_k(\tau _n)&= -0.2\sum _{k=0}^{K} a_k P_k(0) + 0.1\sum _{k=0}^{K} b_k P_k(0) + 0.1\sigma _2\xi _2(t_n). \end{aligned}$$

These equations are linear in $a_k$ and $b_k$, but there will be $N+1$ such equations, one for each collocation point $t_n$. You can solve this system of equations numerically using methods like matrix inversion or iterative solvers to obtain the coefficients $a_k$ and $b_k$ for each *k* in the expansion.

In the Legendre polynomial expansions, we plug these coefficients back into the Legendre polynomial expansions to obtain the approximate solutions *X*(*t*) and *Y*(*t*) over the desired time domain.

Performing the inverse Legendre transform will yield the original time domain values *X*(*t*) and *Y*(*t*). Based on the Legendre polynomial basis functions $a_k$ and $b_k$, the reverse Legendre transform is essentially a weighted sum.

Inverse Transform for *X*(*t*):

$$\begin{aligned} X(t) = \sum _{k=0}^{K} a_k P_k(2t-1). \end{aligned}$$

Inverse Transform for *Y*(*t*):

$$\begin{aligned} Y(t) = \sum _{k=0}^{K} b_k P_k(2t-1). \end{aligned}$$

We calculate *X*(*t*) and *Y*(*t*) at any given time points in the original time domain by substituting the appropriate values of *t* into the above system. Depending on the specific problem and the accuracy required, you may need to truncate the series to a finite number of terms (*K* terms) to approximate the solutions.

Keep in mind that the accuracy of the solutions will depend on the choice of the number of basis functions (*K*) and the collocation points, as well as the precision of the numerical methods used to solve for the coefficients. Adjusting these parameters can help you balance computational efficiency with solution accuracy.

**Figure 1:**
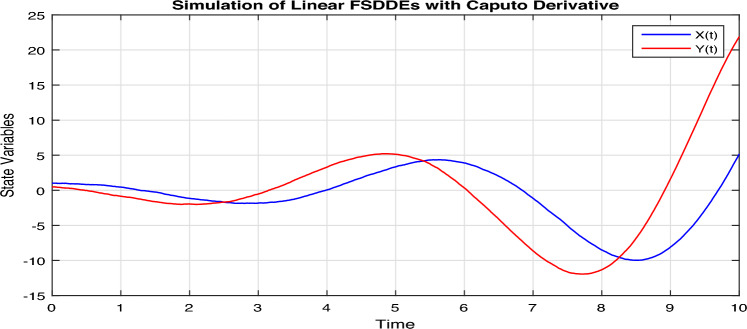
Solution trajectory of FSDDEs system given in (Eq. 14).

**Figure 2:**
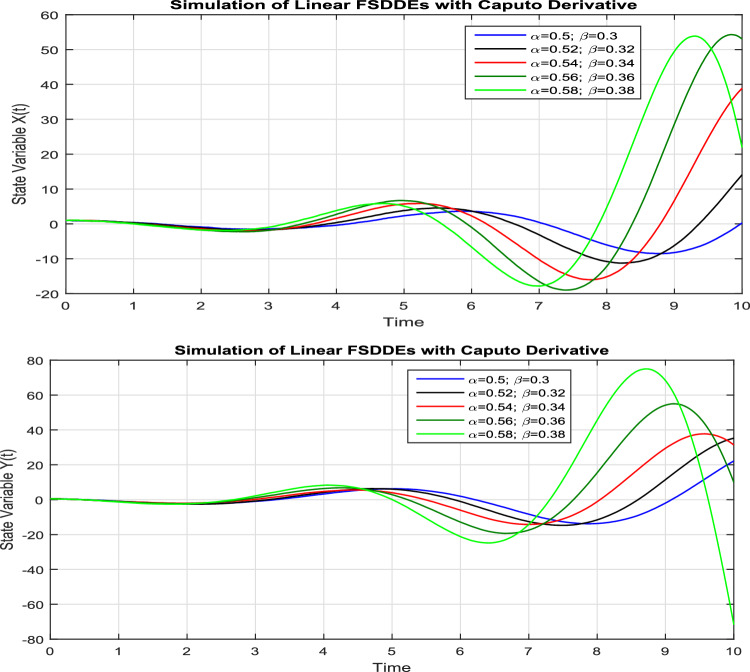
Solution trajectory of FSDDEs system (Eq. 14) for different fractional parameter values $(\alpha =0.5, \beta =0.3), (\alpha =0.52, \beta =0.32), (\alpha =0.54, \beta =0.34), (\alpha =0.56, \beta =0.36), (\alpha =0.58, \beta =0.38)$.

**Figure 3:**
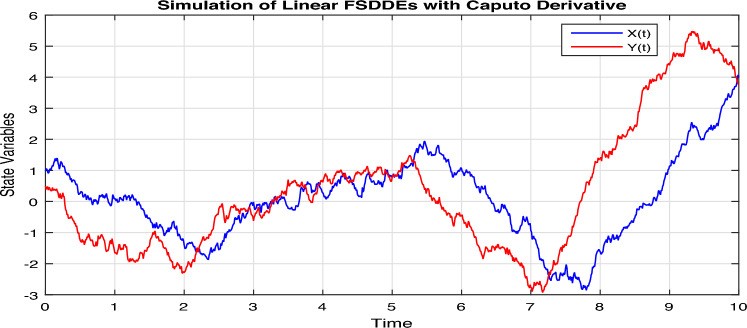
Solution of FSDDEs model given in (Eq. 14) for the stochastic parameter value $\sigma =0.8$.

In Fig. 1, we show the solution trajectory of FSDDEs system given in (Eq. 14) for both *X*(*t*) and *Y*(*t*), using spectral collocation method. For the above simulation, we take the intensity of a standard Brownian motions ($\sigma _1=0.1=\sigma _2$), are very small, therefore we clearly see that the stochastic fluctuation in Fig. 1 is almost negligible. Similarly, in Fig. 2 we show the solution of *X*(*t*) and *Y*(*t*) for different fractional parameter values $\alpha$ and $\beta$, therefore $(\alpha =0.5, \beta =0.3), (\alpha =0.52, \beta =0.32), (\alpha =0.54, \beta =0.34), (\alpha =0.56, \beta =0.36), (\alpha =0.58, \beta =0.38)$. In Fig. 2 we clearly observe the fractional behavior for different fractional parameter values. However, in Fig. 3, we draw the solution of FSDDEs model given in (Eq. 14) for the high stochastic parameter value therefore $\sigma _1=0.8=\sigma _2$. In Fig. 3 we observe that as we increase the stochastic parameters value the fluctuation becomes increases.

### Example 2

*Nonlinear FSDDEs with time-varying coefficients*

Consider a system of nonlinear FSDDEs with time-varying coefficients:

15 $$\begin{aligned} D^{0.7}X(t)&= \sin (t)X(t-\tau ) + 0.1X(t-\tau )^2 + 0.2\sigma _1 \xi _1(t),\nonumber \\ D^{0.5}Y(t)&= \cos (t)Y(t-\tau ) + 0.2Y(t-\tau ) + 0.1\sigma _2 \xi _2(t). \end{aligned}$$

This system has fractional orders $\alpha = 0.7$ and $\beta = 0.5$; where the initial values are $X(0)=1$ and $Y(0)=0.5$. Also the time delay $\tau =1$ and includes stochastic terms.

This system includes nonlinear terms and time-dependent coefficients.

Imagine a chemical reaction in a controlled environment.

The state variable *X*(*t*) could represent the concentration of a chemical species involved in the reaction. The state variable *Y*(*t*) might represent another chemical species. The equations describe how these concentrations change over time.

### Nonlinear effects

The term $\sin (t)X(t-\tau ) + 0.1X(t-\tau )^2$ in the equation for *X*(*t*) introduces nonlinearity. It implies that the rate of change of *X*(*t*) depends not only on its own past values ($X(t-\tau )$) but also on the current time (*t*) and its square ($X(t-\tau )^2$). In a chemical reactions the nonlinearities can arise from complex reaction kinetics, such as inhibitory processes or autocatalytic.

### Time-varying coefficients

These equations have time-varying coefficients, such as $\sin (t)$ and $\cos (t)$. In reaction the rates or other parameters of a given system, there can be changes due to time. However, the chemical reactions may be vary with time due to external or temperature influences changes.

### Fractional orders

In the system Eq. (15), the fractional orders (0.7 and 0.5) shows that the long-range memory effects and dependencies are considered by fractional derivatives. In addition to making the system more capable and realistic for modeling phenomena along with memory effect, the given fractional orders indicates the influence of past states on the current dynamics.

### Stochasticity

The stochastic noise is denoted by $\sigma _1\xi _1(t)$ and $\sigma _2\xi _2(t)$. In chemical reactions the stochasticity or external influences can cause and also contribute to it.

As a result of this example, chemical species concentrations (*X*(*t*) and *Y*(*t*)) evolve over time in a physical system, like a chemical reaction. There is a nonlinear behavior in the system due to complex reaction kinetics and parameters that vary over time, such as reaction rates. Modeling real-world phenomena becomes more complex by adding fractional derivatives and stochastic terms to account for memory effects and random fluctuations.

**Figure 4:**
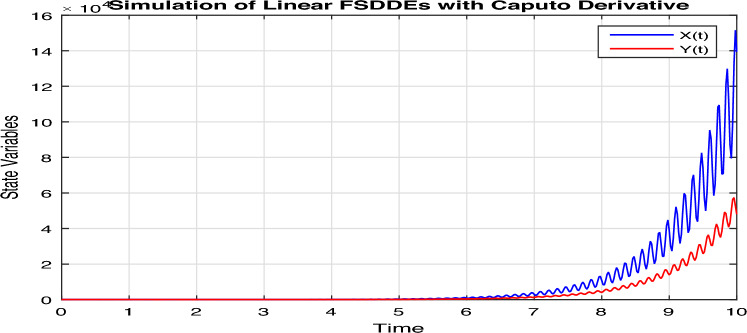
Solution trajectory of FSDDEs system given in (Eq. 15).

**Figure 5:**
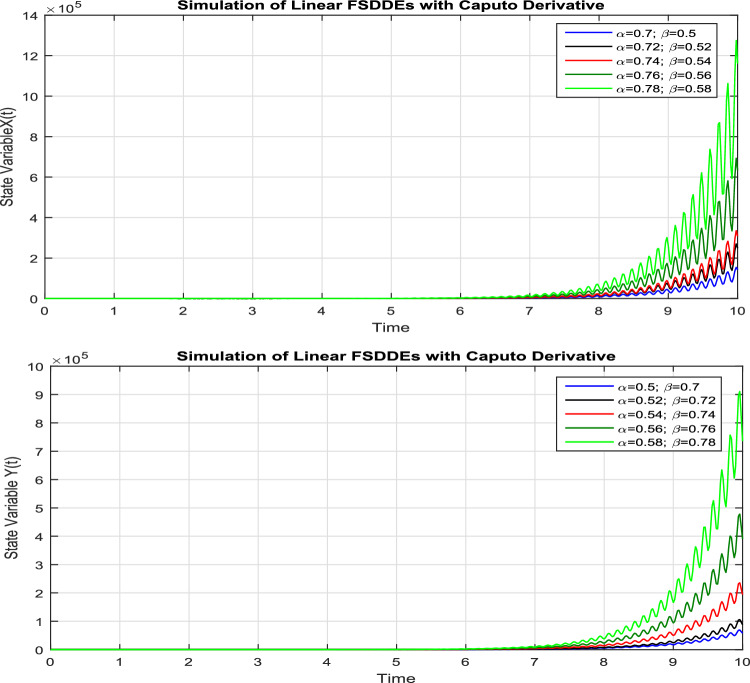
Solution trajectory of FSDDEs system (Eq. 15) for different fractional parameter values $(\alpha =0.7, \beta =0.5), (\alpha =0.72, \beta =0.52), (\alpha =0.74, \beta =0.54), (\alpha =0.76, \beta =0.56), (\alpha =0.78, \beta =0.58)$.

**Figure 6:**
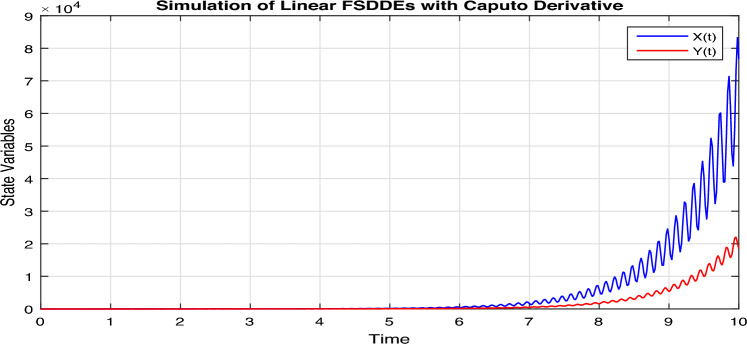
Solution of FSDDEs model given in (Eq. 15) for the stochastic parameter value $\sigma =0.8$.

In Fig. 4, we show the solution trajectory of FSDDEs system given in Eq. (15) for both *X*(*t*) and *Y*(*t*). Similarly, in Fig. 5, we show the solution of *X*(*t*) and *Y*(*t*) for different fractional parameter values $\alpha$ and $\beta$. Also in Fig. 6, we draw the solution of FSDDEs model given in (Eq. 15) for the stochastic parameter value $\sigma =0.8$.

In Fig. 4, we show the solution trajectory of FSDDEs system given in (Eq. 14) for both *X*(*t*) and *Y*(*t*), using spectral collocation method. For the above simulation, we take the intensity of a standard Brownian motions ($\sigma _1=0.1=\sigma _2$), are very small. Similarly, in Fig. 5 we show the solution of *X*(*t*) and *Y*(*t*) for different fractional parameter values $\alpha$ and $\beta$, therefore $(\alpha =0.7, \beta =0.5), (\alpha =0.72, \beta =0.52), (\alpha =0.74, \beta =0.54), (\alpha =0.76, \beta =0.56), (\alpha =0.78, \beta =0.58)$. In Fig. 5, we clearly observe the fractional behavior for different fractional parameter values. Also in Fig. 6, we draw the solution of FSDDEs model given in (Eq. 14) for the high stochastic parameter value therefore $\sigma _1=0.8=\sigma _2$.

#### Example 3

*Fractional diffusion–reaction system*

In this example, we’ll consider a complex system of FSDDEs modeling a fractional diffusion–reaction system:

16 $$\begin{aligned} D^\alpha u(x,t)&= D \frac{\partial ^2}{\partial x^2} u(x,t) - k u(x,t) + \sigma u(x,t) dW(t),\nonumber \\ D^\beta v(x,t)&= D \frac{\partial ^2}{\partial x^2} v(x,t) + k u(x,t) - \lambda v(x,t) + \sigma v(x,t) dW(t). \end{aligned}$$

Here, $u(x,t)$ and $v(x,t)$ represent concentrations of chemical species, $\alpha =0.8$ and $\beta =0.6$ are fractional orders, $D=1$, $k=0.1$, $\sigma =0.4$, and $\lambda =0.2$ are constants, and $dW(t)$ represents Wiener increments in time.

**Figure 7:**
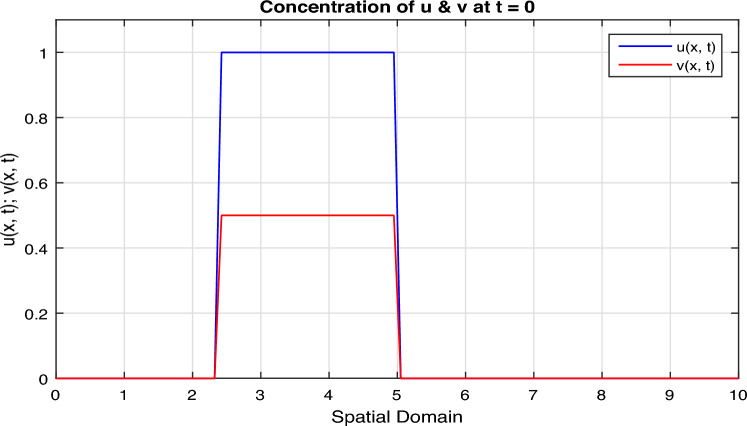
Solution concentration profiles of FSDDEs system given in (Eq. 16) at $t=0$.

**Figure 8:**
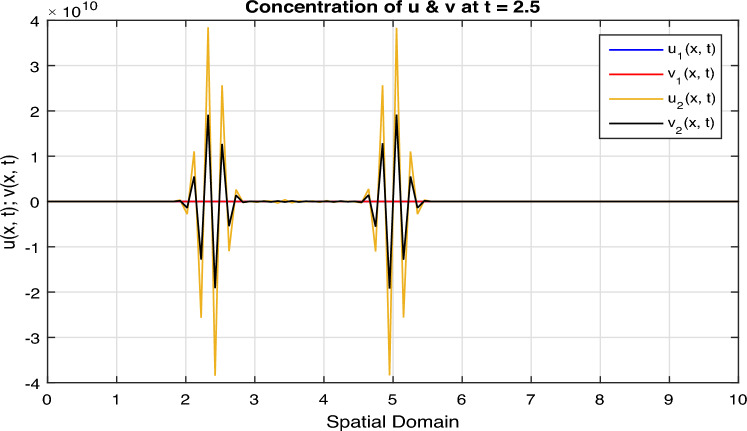
Solution concentration profiles of FSDDEs system given in (Eq. 16) at $t=2$.

**Figure 9:**
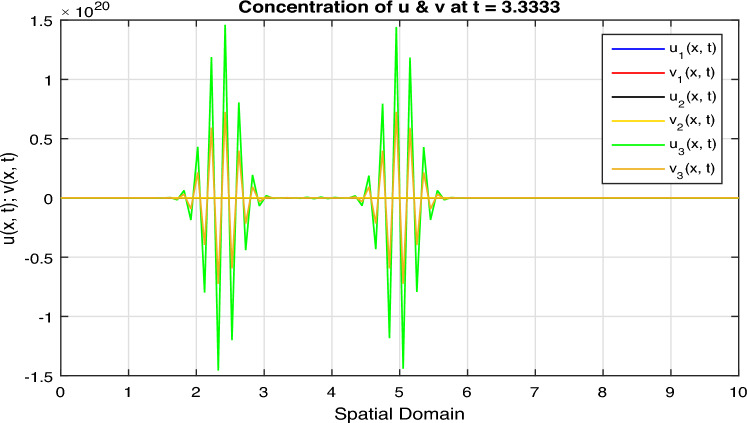
Solution concentration profiles of FSDDEs system given in (Eq. 16) at $t=3$.

**Figure 10:**
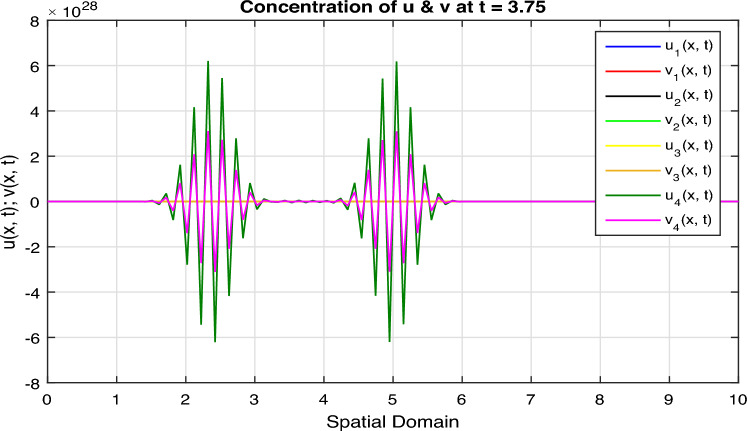
Solution concentration profiles of FSDDEs system given in (Eq. 16) at $t=4$.

**Figure 11:**
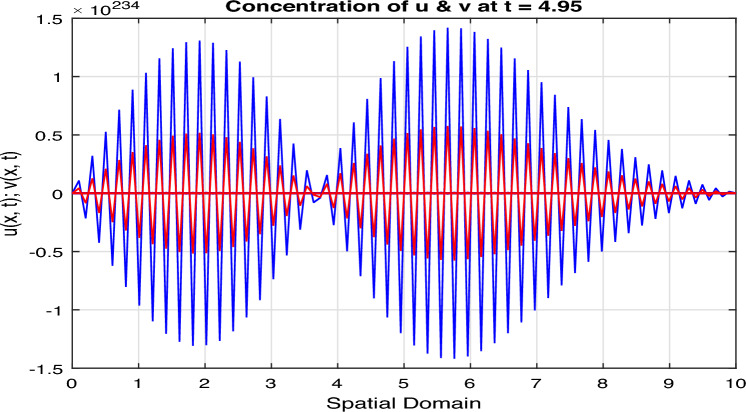
Solution concentration profiles of FSDDEs system given in (Eq. 16) at $t=100$; where blue color for $u_i$ and red color for $v_i, i=0, 1, 2, ...., 100$.

Certainly, let’s discuss the graphs at different time points obtained from the simulation of the Fractional Diffusion–Reaction System using Caputo derivatives. In the provided code, we captured the concentrations of $u$ and $v$ at various time steps. Below is a discussion of these graphs at different $t$ values:

- *t = 0:* In Fig. 7, at the initial time step, $t = 0$, both $u(x, t)$ and $v(x, t)$ start with the specified initial conditions. $u$ has a distribution in the middle of the spatial domain, while $v$ starts with a different distribution.
- *t = 2:* In Fig. 8, as time progresses, $u(x, t)$ diffuses outward from its initial concentration center due to the diffusion term ($D \cdot u_{xx}$), while $v(x, t)$ evolves as a result of the reaction and diffusion terms. $v$ starts to react with $u$ and adjusts its distribution accordingly. In the above Fig. 8, we observe that only the $u_2(x, t)$ and $v_2(x, t)$ graphs are shown and $u_1(x, t)$ and $v_1(x, t)$ graphs are disappear, because the range of $u_1(x, t)$ and $v_1(x, t)$ is 1 see in Fig. 7, while the range of $u_2(x, t)$ and $v_2(x, t)$ is almost $10^{10};$ therefore the smaller range go down and becomes disappear.
- *t = 3:* In Fig. 9, at this time step, the diffusion process continues, and the profiles of both $u$ and $v$ continue to change. The concentration profiles of $u$ and $v$ evolve, reflecting the diffusion and reaction processes in the system. Once again in Fig. 9, we observe that only the $u_3(x, t)$ and $v_3(x, t)$ graphs are shown and the remaining $u_1(x, t)$, $v_1(x, t)$, $u_2(x, t)$ and $v_2(x, t)$ graphs are disappear, because the range of $u_3(x, t)$ and $v_3(x, t)$ is almost $10^{20};$ therefore the graphs having smaller range go down and becomes disappear.
- *t = 4:* In Fig. 10, by $t = 3$, we can observe further spreading of $u(x, t)$ and the impact of the reaction term ($-k \cdot u \cdot v$) on both $u$ and $v$. The distribution of $v$ adapts to the presence of $u$. Again in Fig. 10, we see the range of $u_4(x, t)$ and $v_4(x, t)$ is almost $10^{28};$ therefore the remaining graphs $u_3(x, t)$ and $v_3(x, t)$, $u_2(x, t)$ and $v_2(x, t)$ and $u_1(x, t)$ and $v_1(x, t)$ having smaller range therefore they go down and becomes disappear.
- *t = 100:* In Fig. 11, at this point, $u(x, t)$ and $v(x, t)$ continue to evolve. The reaction between $u$ and $v$ becomes more prominent, leading to changes in their concentration profiles. We clearly observed from Fig. 11, that if we increase the time step, both the chemical species $u$ and $v$ increase the concentration with each other. The impact of stochastic noise ($\sigma \cdot \text {randn}$) also plays a role in the variability of the profiles.

These discussions highlight the dynamic behavior of the system over time. As time progresses, the diffusion, reaction, and stochastic terms interact to shape the concentrations of $u$ and $v$. The plots provide insights into the spatiotemporal evolution of the chemical species in the system, showing how they spread, react, and adapt to each other and to external noise.

Solving such complex systems typically requires dedicated numerical libraries and computational resources, as well as a deep understanding of both fractional calculus and stochastic processes.

## Challenges and future directions

Fractional stochastic systems pose challenges in terms of analytical solutions and numerical simulations due to the combination of non-integer derivatives and stochastic components. However, magnifications in each computational techniques, that is Monte Carlo simulations, spectral methods, and for fractional calculus the numerical algorithms have made it possible to simulate and analyze such systems with high accuracy. In summery, the fractional stochastic systems provide a powerful techniques for such complex dynamics modeling that exhibit both stochastic characteristics and fractional calculus. The proposed systems having applications in a continue to be a subject of active research and wide range of fields, offering perception into the real-world phenomena’s behavior of that conventional models may struggle to capture.

## Conclusion

In this research work, we have precisely introduced a novel mathematical approach for a systems constrained by FSDDEs and their stability analysis using the spectral collocation technique a strong mathematical approach. By combining the power of spectral techniques, stochastic processes, and fractional calculus, this study offers expensive perception into the stability features of complex dynamical systems along with delays. Numerical simulations confirm the efficiency and accuracy of our approach, for researchers making it a valuable, practitioners working and worthy tool with FSDDEs in various scientific research fields. This knowledge presents substantially for real-world phenomena to our understanding, offering a foundation for predicting and controlling systems exhibiting stochastic dynamics and along with fractional derivative. This research gives the way for further applications and exploration in different fields, from finance to biology. In future this research work is extended to the analysis to more complex systems and investigate the additional applications in engineering and science.

## Data Availability

The datasets used and/or analysed during this study available from the corresponding author on reasonable request.
